# Unravelling transmission ratio distortion across the bovine genome: identification of candidate regions for reproduction defects

**DOI:** 10.1186/s12864-023-09455-6

**Published:** 2023-07-08

**Authors:** Samir Id-Lahoucine, Joaquim Casellas, Aroa Suárez-Vega, Pablo A. S. Fonseca, Flavio S. Schenkel, Mehdi Sargolzaei, Angela Cánovas

**Affiliations:** 1 Centre for Genetic Improvement of Livestock, Department of Animal Biosciences, University of Guelph, Guelph, ON N1G 2W1 Canada; 2 Departament de Ciència Animal I Dels Aliments, Universitat Autònoma de Barcelona, Bellaterra, 08193 Barcelona, Spain; 3 Department of Pathobiology, University of Guelph, Guelph, ON N1G 2W1 Canada; 4 Select Sires, Inc, Plain City, OH 43064 USA

**Keywords:** Transmission ratio distortion, Cattle Genomics, Reproduction, Mendelian inheritance, Deleterious mutations

## Abstract

**Background:**

Biological mechanisms affecting gametogenesis, embryo development and postnatal viability have the potential to alter Mendelian inheritance expectations resulting in observable transmission ratio distortion (TRD). Although the discovery of TRD cases have been around for a long time, the current widespread and growing use of DNA technologies in the livestock industry provides a valuable resource of large genomic data with parent–offspring genotyped trios, enabling the implementation of TRD approach. In this research, the objective is to investigate TRD using SNP-by-SNP and sliding windows approaches on 441,802 genotyped Holstein cattle and 132,991 (or 47,910 phased) autosomal SNPs.

**Results:**

The TRD was characterized using allelic and genotypic parameterizations. Across the whole genome a total of 604 chromosomal regions showed strong significant TRD. Most (85%) of the regions presented an allelic TRD pattern with an under-representation (reduced viability) of carrier (heterozygous) offspring or with the complete or quasi-complete absence (lethality) for homozygous individuals. On the other hand, the remaining regions with genotypic TRD patterns exhibited the classical recessive inheritance or either an excess or deficiency of heterozygote offspring. Among them, the number of most relevant novel regions with strong allelic and recessive TRD patterns were 10 and 5, respectively. In addition, functional analyses revealed candidate genes regulating key biological processes associated with embryonic development and survival, DNA repair and meiotic processes, among others, providing additional biological evidence of TRD findings.

**Conclusions:**

Our results revealed the importance of implementing different TRD parameterizations to capture all types of distortions and to determine the corresponding inheritance pattern. Novel candidate genomic regions containing lethal alleles and genes with functional and biological consequences on fertility and pre- and post-natal viability were also identified, providing opportunities for improving breeding success in cattle.

**Supplementary Information:**

The online version contains supplementary material available at 10.1186/s12864-023-09455-6.

## Background

Exceptions to ordinary Mendelian principles, as the non-random transmission of alleles from parents to offspring, have been described in both model and non-model organisms (e.g., [1–4]. This phenomenon, where Mendelian inheritance expectations are altered (regardless of the cause), is known as transmission ratio distortion (TRD, [5]. From a biological point of view, a wide variety of mechanisms that affect germ cells (e.g., meiotic drive, germline selection, gametic competition, [6], embryo lethality [7], and even differential postnatal viability [8] have the potential to violate Mendel’s law of segregation. Although TRD remains an unclear and ambiguous phenomenon in the scientific literature [9, 10], it can be considered the ultimate outcome of several genetic factors that arise at different stages of the reproductive process and early neonatal life. In fact, particular cases of TRD, such as the absence of homozygosity, are already being used to target recessive defects that affect reproduction (e.g., decreased fertility, embryo loss) in different livestock species [11, 12]. Indeed, the absence of homozygous offspring may also be attributed to inbreeding depression, which is a potential source of TRD [13]. Nowadays, the rapid development of high-throughput genotyping technologies and the increasing number of genotyped animals, with over 3,000,000 Holstein genotypes in North America alone [14], has opened alternative genomic strategies to improve reproductive efficiency. This is crucial because reproductive defects and genetic abnormalities are prevalent and have a significant impact on animal productivity. The TRD approach, which requires only genomic information without phenotypic records, aims to be an applicable strategy with potential outcomes to improve reproductive success in livestock [15]. Within this context, the aim of this study is to investigate TRD in Holstein cattle with the hypothesis that novel chromosomal regions containing lethal alleles or potential genes affecting reproduction can be discovered. This will provide valuable insights into the genetic background of the reproduction.

In this study, we focused on implementing different TRD models based on tracing allele inheritance from parents to offspring using parent–offspring genotype trios of Holstein cattle. The TRD models derived from two parameterizations: (i) allelic parameterization with specific sire- and dam-TRD, or merging both into one overall TRD [15, 16], and (ii) genotypic parameterization modeling interaction between alleles in offspring genotypes, including additive and dominance components of TRD [17]. Therefore, the main objectives of this study were (1) to describe, interpret and compare TRD parameterizations and to highlight their differences and potential applications, (2) to present a comprehensive characterization of TRD across the entire genome of Holstein cattle, (3) to assess the inheritance pattern of regions with known lethal alleles in order to determine the reliability of the TRD approach, (4) to identify novel genomic regions with moderate to high TRD penetrance potential to harbor deleterious mutations, and (5) to annotate genes and to evaluate the functional consequences of TRD regions using functional analyses.

## Results and discussion

### Prevalence of TRD across Holstein genome

Decisive evidence (Bayes factor (BF) ≥ 100) according to Jeffreys’ scale [18] was identified for TRD in both individual SNPs and haplotypes across the Holstein genome. All the post TRD analyses criteria were implemented to minimize and discard TRD artifacts coming from genotyping errors and random TRD given the sample size of informative offspring. Particularly, the specific approximate empirical null distributions of additive- and dominance-TRD used to discard random TRD from the genotypic model were developed following [19] and summarized in the supplementary Material 1. After applying the previously given filtering criteria, 271 and 700 SNPs were identified with distorted segregation from SNP-by-SNP analyses in raw- and imputed-data, respectively. Using imputed data for haplotype analyses, the total numbers of allele-regions with TRD were 3,115, 8,566, 17,558, 25,638 and 39,433 for 2-, 4-, 7-, 10- and 20-SNP haplotypes, respectively. The potential of capturing more signals of TRD with haplotype-based method is given its ability to exploit the available SNPs and provide additional range of allele frequencies across the whole genome as reported by Id-Lahoucine et al. [19] in more details. Among these findings, it is worth highlighting that the majority of regions were detected with more than one of the models applied (i.e., parent-unspecific model, parent-specific model and genotypic model). Only 3.06% and 2.49% regions were identified uniquely on parent-specific or genotypic TRD models, respectively. Despite this overlap, different statistical significance values were obtained for TRD estimates, suggesting different degrees of fit among the models. After exclusively keeping the allele-region (i.e., a SNP or a haplotype from a window) with the highest BF within a region, 51,364 regions were identified (including totally or partially overlapped windows). This reported initial number of regions can be considered reasonable for TRD phenomenon in comparison to results in Hoff et al. [12], who reported 12,020 haplotypes of 20-SNP with absence of homozygosity in 3,993 genotyped animals and a minor allele frequency (MAF) of 0.02.

#### Rare variants identified by TRD approach

Most of the regions detected with TRD presented low frequencies and were supported by the large dataset. Among them, 47,839, 44,424, 40,358, 36,864, 26,419, 18,516 and 5 regions had a MAF < 0.05, 0.02, 0.01, 0.005, 0.001, 0.0005 and 0.0001, respectively. These findings, in contrast to other methodologies, revealed the TRD approach as a very powerful method to detect rare variants. In fact, it has been suggested that targeting rare variants is more power to detect causal mutations than common variants [20]. Moreover, Ghanem and Nishibori [21] reported that most of the recessive variants associated with a decline in fertility and pregnancy losses in cattle are difficult to discover and may not be detected.

### Integration of TRD mapping

**Fig. 1:**
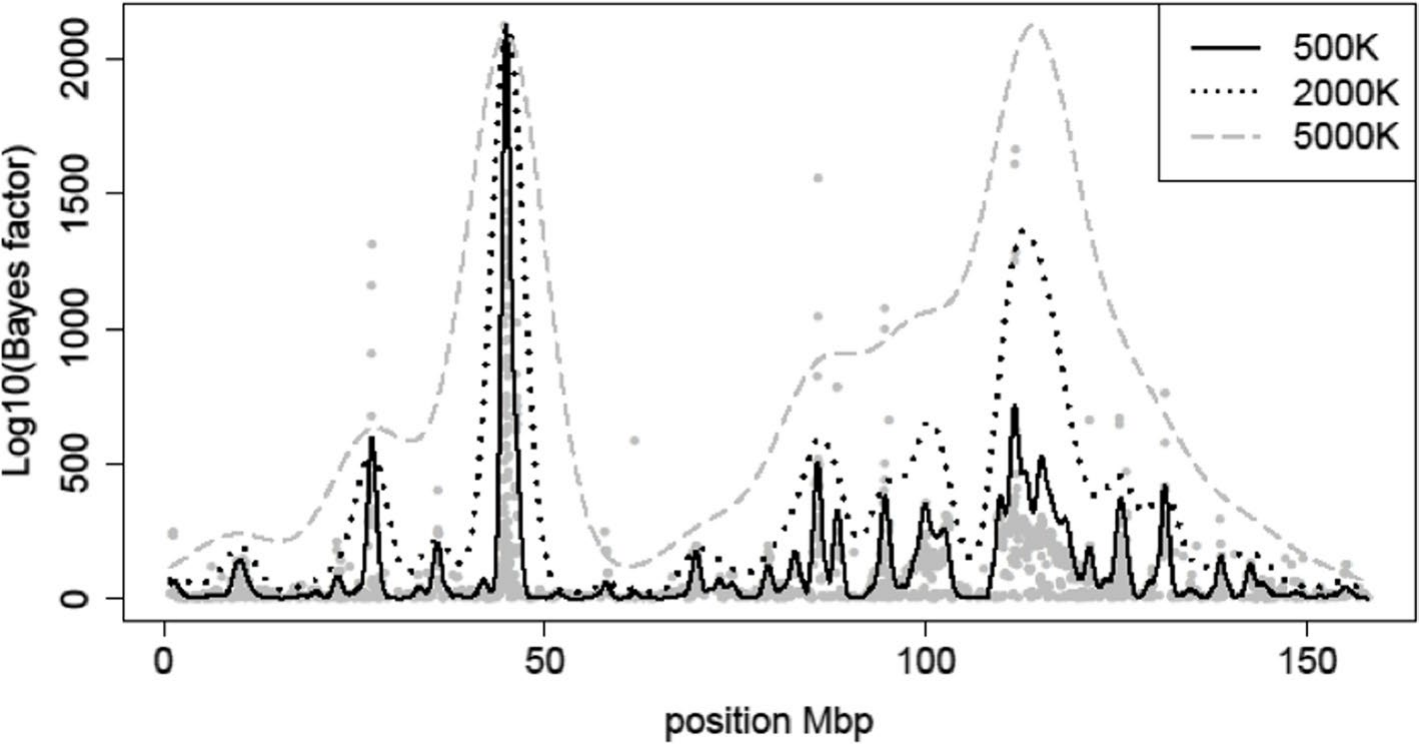
Bayes factor for transmission ratio distortion in BTA1 with Kernel smoothing using different bandwidth (i.e., 500,000, 2,000,000 and 5,000,000 bp)

A total of 51,364 regions were identified across the whole genome. This high number of regions is a result of different features, including the sliding window approach used, the different window sizes implemented, the level of linkage disequilibrium (LD) and the patterns of TRD observed for individual SNPs (or short haplotypes), which are also displayed across the haplotypes that include them. We assumed that the different patterns observed across adjacent regions were generated from one single mutation that underlies the observed TRD. The allele-region with the highest BF was assumed to be the best candidate region harboring the causal variant or in strong LD with it. This assumption was made taking into consideration that the BF simultaneously combines both the magnitude of TRD and the number of informative offspring [19]. After the integration of LD with the smoothing process in order to obtain clear highlighted peaks of TRD across the whole genome, 797 core regions were differentially identified. This result was based on bandwidth of 500,000 bp for the smoothing process, which was assumed as a sensible distance to obtain a considerable initial number of candidate regions. Notice that the choice of the bandwidth for smoothing is an important component of its implementation. Figure 1 shows the different patterns of kernel smoothing for several fixed bandwidths across a single chromosome with the rescaled smoothed BF. Here, with bandwidth of 500,000 bp a specific region was assumed to show significant TRD at 95, 99 and 99.9% up to ± 980,000, ± 1,290,000 and ± 1,645,000 bp, respectively. Among the obtained 797 core regions after the smoothing process, 193 regions were excluded as they were plausibly explained by genotyping errors after individually checking the parameters estimated and the corresponding distribution of the offspring across matings. Supplementary Material 2 summarizes the information for the final 604 chromosomal regions deemed with TRD.

### Goodness-of-fit and pattern of TRD

Multiple mechanisms from gamete formation in the parental generation to offspring viability can cause TRD. In order to differentiate among the various potential causes, Pardo-Manuel de Villena et al. [5] developed a test to discriminate between meiotic and postmeiotic mechanisms of TRD based on the recombinational status between the centromere and the distorted locus. Leppälä et al. [22] used a likelihood ratio test to distinguish between gametic or zygotic TRD in classical experimental designs of F2 crosses. The different models developed by Casellas et al. 2012, 16, 15, 18, arising from different complementary parameterizations, can determine the genetic mechanism (or inheritance pattern) involved in a region and even provide additional evidence on the level where the TRD is likely to be [23].

When comparing different TRD models across the Holstein genome, different goodness-of-fit in terms of deviance information criterion (DIC) were observed. Models with smaller DIC values indicate a better fit, and differences between models greater than 3 DIC units are considered statistically relevant [24]. For allelic parameterization, in the absence of parent-specific TRD, similar magnitudes of TRD effects (i.e., overall, sire- and dam-TRD) and similar fit with minimal differences in DIC units between the parent-unspecific and -specific models, were observed, supporting the overall TRD pattern. However, under a parent-specific TRD pattern, differences from 3 up to 599 DIC units were observed between the allelic models (i.e., parent-unspecific and -specific TRD). On the other hand, the genotypic model was favored in certain genomic regions reaching reductions up to 6,442.85 DIC units in relation to the allelic model.

Different TRD models were clearly favored by DIC among TRD regions suggesting the corresponding inheritance pattern of the observed TRD. Within this context, among 604 identified regions, 195 fit better to the allelic model with overall TRD. When the parent-specific model displayed minimal DIC units, 91 regions exhibited sire-TRD (null via dam), 6 showed dam-TRD (null via sire) whereas 220 regions presented both sire- and dam-TRD with different magnitudes (i.e., different penetrance or LD among them). Finally, the genotypic model fit better for 92 regions highlighting the corresponding additive and dominance components of TRD.

### Allelic versus genotypic patterns

**Fig. 2:**
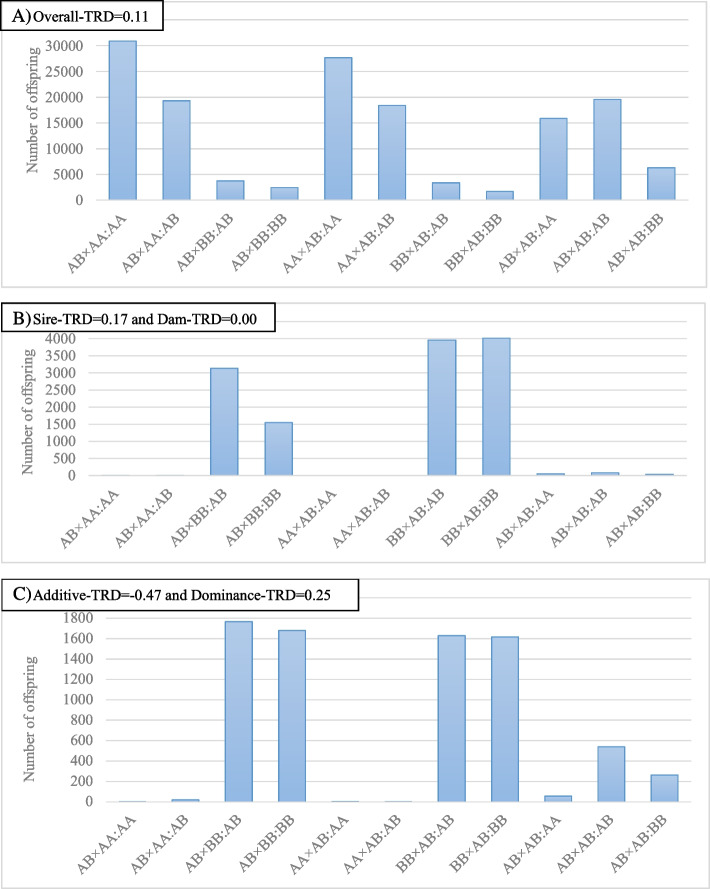
Distribution of offspring across matings (Sire × Dam:Offpsring) from different regions with transmission ratio distortion

From a biological point of view, the allelic parameterization, which focuses on a single-allele basis, is more likely to be associated with TRD occurring prior to fertilization (i.e., haploid phase), whereas the genotypic parameterization is more appropriate for events occurring after fertilization given its basis on the (combined) genotype itself (i.e., during the diploid phase; [23]. However, the peculiarity of TRD findings on real data, such as the low frequency of alleles on TRD regions, must be taken into consideration. Most of the identified TRD regions showed a lack of some types of matings and an imbalanced number of trios. Thus, a relatively few (or none) heterozygous-by-heterozygous mating and the absence or near absence of homozygous individuals in both parental and offspring generations further supports deleterious effects of the identified TRD regions. Nevertheless, at the biological level, the imbalanced number of trios among matings or the absence of specific matings could make it difficult to differentiate between allelic and genotypic related TRD phenomenon [23]. Therefore, although most regions detected showed allelic patterns (~ 85%), this does not ensure their occurrence before fertilization. In fact, functional and positional evidence for regions with the allelic pattern suggested that biological related events act both pre-and post-fertilization. Further, the presence or absence of a specific allele, independently of the homologous one, may be sufficient to induce lethality and consequently generate TRD. Moreover, taking into consideration the moderate-to-high correlation between the overall TRD and additive-TRD, the allelic patterns could also be viewed as an additive effect in the offspring genotype, where the presence of the allele gives a dosage effect, reducing the viability of carrier offspring. It must be noted that an important part of regions with allelic pattern showed complete or quasi-complete absence (lethality) for homozygous individuals, but also an under-representation (reduced viability) of heterozygous offspring. Indeed, Khatib et al. [25] described a similar pattern of distortion in a fertility candidate gene study. These observed patterns across the Holstein genome emphasize the importance of the allelic parameterization. Table 1 and Fig. 2 illustrates the distribution of offspring for each type of mating showing different patterns of TRD with the corresponding TRD estimates for the fitted model.

**Table 1:** Distribution of offspring from all matings of distorted regions with different transmission ratio distortion (TRD) pattern and corresponding TRD estimates

TRD effects	AB × AA^a^		AB × BB		AA × AB		BB × AB		AB × AB		
	AA^b^	AB	AB	BB	AA	AB	AB	BB	AA	AB	BB
AL α = 0.11	30876	19298	3725	2431	27698	18405	3341	1690	15876	19540	6261
AL α = -0.42	0	0	117	1671	0	0	135	1347	0	2	8
AL α_s_ = 0.23 α_d_ = 0.15	40014	14981	161	78	20154	11232	125	39	4787	3826	998
AL α_s_ = -0.43 α_d_ = -0.16	0	0	611	8293	0	0	1055	1996	8	25	66
AL α_s_ = -0.34 α_d_ = -0.17	0	0	2402	12608	0	0	3039	6350	25	315	346
AL α_s_ = -0.38 α_d_ = -0.01^c^	0	0	143	1042	0	0	680	702	2	4	6
AL α_s_ = 0.17 α_d_ = 0.00^c^	1	1	3135	1545	0	0	3958	4019	47	79	36
AL α_s_ = 0.02^c^ α_d_ = -0.09	0	0	1508	1400	0	0	997	1440	0	8	7
GN^d^ α_g_ = -0.66 δ_g_ = 0.33	0	0	5163	5226	0	0	6967	6907	0	259	118
GN^d^ α_g_ = -0.47 δ_g_ = 0.25	1	22	1767	1679	4	2	1630	1617	58	539	264
GN^e^ α_g_ = 0.09 δ_g_ = -0.04	26929	23757	2234	2312	36680	31499	1175	1180	9175	14625	7262
GN^f^ α_g_ = -0.05 δ_g_ = 0.04	18436	21261	13813	13452	22549	26321	12,587	12607	16140	36341	17488
GN^g^ α_g_ = 0.05 δ_g_ = 0.25	14472	19113	17553	10166	18857	28751	9198	5534	20075	70790	18453
GN^h^ α_g_ = -0.40 δ_g_ = -0.25	25176	20055	9001	15901	10972	10863	5519	10,268	14901	26163	32008

*AL* Allelic, *GN* Genotypic, α Overall TRD, α_s_ Sire-TRD, α_d_ Dam-TRD, α_g_ Additive-TRD, δ_g_: Dominance-TRD

^a^Sire × dam mating genotypes

^b^Offpsring genotype from the corresponding mating

^c^Non-significant

^d^Recessive pattern

^e^Homozygote advantage

^f^Homozygote disadvantage

^g^Heterosis excess

^h^Heterosis deficiency

On the other hand, the genotypic model was sensitive to the lack of some type of matings and the imbalance in number of trios on TRD estimations. Under these conditions, TRD estimates must be interpreted with caution, as they could be less accurate due to the ability of the model to converge to a combination of additive- and dominance-TRD that maximize the imbalance of matings. Nevertheless, when a clear interaction between alleles is observed and supported by the distribution of the offspring genotypes, this could substantiate the post fertilization events of the observed TRD. For example, the genotypic model highlighted regions with the classical recessive patterns, in these cases, homozygous offspring were not observed from heterozygous-by-heterozygous matings, whereas the theoretical Mendelian ratio (i.e., null TRD) was maintained on heterozygous-by-homozygous matings. Recessive haplotypes as Holstein haplotype 0 (HH0, [26, 27], haplotype 1 (HH1, [11, 28], haplotype 3 (HH3, [11, 29] and haplotype 5 (HH5, [30], which are known to be due to embryonic lethality [21, 31], were also identified with a genotypic recessive pattern when analyzed by TRD approach presented here (Table 2). Other patterns found with the genotypic model can be related to heterosis effect. In these regions, an excess or deficiency of heterozygous offspring was observed (Table 1). A similar pattern of heterozygote excess was also observed in plants [9]. We cannot rule out that the mentioned heterozygote advantage may come from a parent-specific TRD, where different alleles have opposite preferential transmissions, resulting in an over-representation of heterozygous offspring.

**Table 2:** Patterns of transmission ratio distortion (TRD) of previously described recessive haplotypes

Breed	Haplotype	Chromo- some	Region1 (Mbp)^a^	Region2 (Mbp)^b^	Frequency %	AA × AB^e^		AB × BB		AB × AB			TRD effects
						AA^f^	AB	AB	BB	AA	AB	BB	
Holstein	HH3 (7)^g^	8	95.4	94.8–95.7	2.830	0	0	12130	12133	0	259	118	α_g_ = -0.66, δ_g_ = 0.33
	HH2 (9)	1	94.8–96.5	94.1–94.6	0.411	0	0	1224	3027	1	6	17	α = -0.21, α_s_ = -0.29, α_d_ = -0.15
	HH1 (23)	5	63.1	62.6–63.2	0.949	0	0	3809	3669	0	30	14	α_g_ = -0.60, δ_g_ = 0.32
	HH0 (22)	21	21.1–21.1	20.2–21.2	1.565	0	0	6256	6248	0	69	43	α_g_ = -0.64, δ_g_ = 0.32
	HH4	1	1.2	1.0–1.3	0.061	0	0	301	1583	0	1	6	α = -0.34, α_s_ = -0.42, α_d_ = -0.11
	HH5	9	93.2–93.3	92.2–92.4	2.69	1	5	14729	15184	10	355	166	α_g_ = -0.62, δ_g_ = 0.29
	HHB	1	145.1	142.5–142.5	1.115	2	2	5175	8063	28	72	44	α = -0.11, α_g_ = -0.33, δ_g_ = -0.09
	HHC (23)	3	43.4	44.8	55.470	40985	47582	26400	26059	16140	36341	17488	α_g_ = -0.05, δ_g_ = 0.04
	HCD	11	77.9	77.0–78.0	10.679	357	451	20681	26386	1254	3482	1989	α = -0.06, α_g_ = -0.25, δ_g_ = -0.01
		18	50–60	58.5–58.7	0.15	0	0	575	6770	0	1	1	α = -0.42, α_s_ = -0.44, α_d_ = -0.21
		18	50–60	61.2	87.30	5172	5263	5	0	509	1246	61	α_g_ = 0.56, δ_g_ = 0.30
Jersey	JH2	26	8.8–9.4	9.0–12.2	0.001	0	0	603	1424	0	0	0	α = -0.20, α_s_ = -0.21, α_d_ = -0.00
	JH1 (90)	15	15.7	15.0–15.1	0.183	0	0	897	1508	3	1	3	α = -0.13, α_s_ = -0.15, α_d_ = -0.11
Brown Swiss	BH2 (14)	19	11.0	10.1–11.7	0.078	0	0	451	1332	0	0	0	α = -0.25
	BHM (3)	24	62.1–62.2	61.6–61.8	0.041	0	0	55	921	0	0	2	α = -0.44
Angus		1	27.7–29.0	27.2–27.3	0.380	0	0	1204	8175	1	20	132	α = -0.37, α_s_ = -0.44, α_d_ = -0.30
		29	43.0–44.2	34.6–34.7	0.676	0	0	3461	10560	15	46	62	α = -0.25, α_s_ = -0.30, α_d_ = -0.15

α Overall TRD, α_s_ Sire-TRD, α_d_ Dam-TRD, α_g_ Additive-TRD, δ_g_ Dominance-TRD

^a^The original coordinates of the causal variant, gene or the haplotype (http://aipl.arsusda.gov/reference/recessive_haplotypes_ARR-G3.html)

^b^The coordinates for SNPs or haplotype windows with TRD

^c^Number of SNPs on haplotype window

^d^Number of heterozygous sires or dams

^e^Parents’ genotypes

^f^Offpsring genotype

^g^Number of unobserved expected homozygous offspring

In general, independently of the moment when the TRD occurred, both parameterizations showed to be complementary, allowing to capture different types of TRD, emphasizing the importance of implementing the three models. It is remarkable that the prevalence of regions with recessive TRD patterns is small in comparison to allelic TRD regions, which may potentially explain part of the reproductive inefficiency of the animals. Embryo losses during the first 42 days range from 30 to 40% in most domestic species [32] and average calving rates after single insemination are 57.5% and 37.5% for heifers and lactating Holstein cows, respectively [33], which exemplify reproductive inefficiencies that might be related to allelic TRD regions. In addition, it is worthy to highlight that the implementation of full trios allowed clear differentiation between recessive and allelic patterns, as heterozygous-by-heterozygous matings are necessary to predict the lethality of homozygous genotypes. Notice that the few or none of the trios of the heterozygous-by-heterozygous matings of the detected regions, in addition to their law chance given the low frequency, it could also be partially explained by producers’ mating decisions to avoid inbreeding.

### Examining the inheritance patterns of the previously described lethal haplotypes

The inheritance patterns of already published regions with lethal alleles were examined to validate the finding of the TRD approach. Table 2 summarizes the previously describe recessive haplotype regions with TRD findings (additional results are presented in Supplementary Material 3).

#### Inheritance pattern of Holstein lethal haplotypes

HH0 [26], HH1 [11, 28] and HH3 [11, 29] were described previously by VanRaden et al. [11] with 22, 23 and 7 non-observed homozygous offspring from heterozygous carrier sires with heterozygous carrier maternal grandsire matings (Table 2), respectively. Haplotype-windows with recessive patterns were detected by the genotypic model using TRD approach covering HH0 haplotype or the corresponding causal mutation itself for HH1 and HH3 haplotypes. TRD corresponding results showed 43, 14 and 118 non-observed homozygous offspring from double heterozygous mating, respectively (Table 2). A haplotype displaying a genotypic TRD pattern with 156 non-observed homozygous offspring was also detected at ~ 800 Kbp from HH5 [30]. These 4 recessive Holstein haplotypes were identified displaying an additive-TRD ranging from -0.60 to -0.66 and their unfavorable effects were minimized by a favorable dominance-TRD effects (ranging from 0.29 to 0.33) in heterozygous offspring, allowing for their survival. Holstein haplotype 2 (HH2, [11, 29], haplotype 4 (HH4, [34], bovine leukocyte adhesion deficiency (HHB, [35], deficiency of uridine monophosphate synthase (HHD, [36] and Holstein cholesterol deficiency (HCD, [30] regions were also identified as exhibiting significant signals of TRD with allelic patterns. These were not only detected observing the absence (or relative absence) of homozygous offspring, but also an important reduced number of heterozygous offspring from heterozygous-by-homozygous matings (Table 2, Supplementary Material 3). For the complex vertebral malformation (HHC, [37], alleles across this region were identified with either an allelic or genotypic pattern. The genotypic pattern showed an interaction between alleles with a disadvantage for homozygous haplotypes (α_g_ = -0.05 and δ_g_ = 0.04,Table 2, Supplementary Material 3). Moreover, a SNP (BTA18:61,231,528) and a haplotype (BTA18:58,551,307–58,696,066) of TRD regions were observed to be physically close to a QTL with a major effect on various fertility traits, such as calving ease and stillbirth. These QTL were reported by various studies during the last decades (e.g., [38, 39] and reviewed and confirmed by Müller et al. [40] and investigated at sequence level more recently by Dachs et al. [41]. In our TRD results, the haplotype (BTA18:58,551,307–58,696,066) was detected with an allelic pattern and more strongly via sire (α_s_ = -0.44) than dam (α_d_ = -0.21) and the SNP (BTA18:61,231,528) showed a recessive TRD pattern with 448 non-observed homozygous offspring less than expected. Furthermore, from an economic perspective, it is worth noting from that the annual loss attributed to lethal alleles in Holstein cattle has been estimated to be $7,500,265 in the US [31]. This highlights the significance of our research in identifying novel lethal alleles and improving the fertility and reproductive performance of Holstein cattle.

#### Concordance of TRD findings across breeds

When examining regions with known reproduction defects discovered in other cattle breeds, patterns of TRD were also identified in the Holstein data analyzed in this study. Jersey haplotype 1 (JH1; [11, 42] and Jersey haplotype 2 (JH2, [43] were identified with haplotype alleles displaying overall TRD of -0.13 and -0.15, respectively. Brown Swiss haplotype 1 (BH1, [11], Brown Swiss haplotype 2 (BH2, [11, 44], spinal muscular atrophy (BHM, [45, 46], bovine progressive degenerative myeloencephalopathy or weaver (BHW, [47, 48] and spinal dysmyelination (BHD, [49, 50] were identified with haplotype alleles displaying overall TRD of -0.46, -0.25, -0.44, -0.03 and -0.24, respectively (Table 2, Supplementary Material 3). The Ayrshire haplotype 1 (AH1, [51, 52] was also identified, with a haplotype displaying an α = -0.33. Finally, among the candidate lethal haplotypes described with absent homozygosity in Angus cattle by Hoff et al. [12], signals of TRD were also observed, overall TRD ranged from -0.17 to -0.41 on these regions (Table 2, Supplementary Material 3). All theses findings suggest, with the assumption of no recent common ancestor between these breeds and Holstein, that probably independent mutations occurred in the same genes which resulted in generated signals of TRD in the same regions, and consequently, may support the biological function of those genes on reproduction-related traits. In addition, to the best of our knowledge, there have been no investigations on these regions in Holstein cattle to date.

### Novel candidate lethal allele regions

The ultimate goal of this study was to discover novel candidate lethal alleles, genes and even causal mutations directly affecting reproduction and survival. For this, potential regions were chosen to take into consideration the number of under-represented offspring and the magnitude of TRD itself. The number of under-represented offspring, considering the allelic model, is approximately equal to the number of informative offspring multiplied by twice the TRD magnitude, which also corresponds to the sum of the differences between the offspring genotypes within matings. In this context, if one assumes embryonic lethality to be the cause of a specific TRD region, the number of under-represented offspring could be viewed as the possible number of embryo losses. Thus, among the TRD findings, the number of regions with ≥ 1,000 under-represented offspring were 330 and reduced to 146, 102, 69, 35 and 11 when considering ≥ 2,500, 5,000, 10,000, 20,000 and 30,000 under-represented offspring.

On the other hand, as already introduced by Id-Lahoucine et al. [19], the magnitude of TRD describes the probability of an allele being transmitted to viable offspring suggesting its corresponding penetrance. If embryonic lethality is considered as the potential cause of observed TRD, then the probability of observing embryo losses will increase as the magnitude of TRD increases. Therefore, the usefulness of regions with strong TRD (e.g., SNP with high LD with causal mutation or haplotype harboring the causal mutation) for breeding purposes is greater compared to those with lower TRD magnitudes. Furthermore, it is worth noting that the TRD magnitudes are estimated based on population-level data. Therefore, it is possible that both low and moderate TRD signals are associated with specific causal mutations that have been generated and spread within particular families, and where further research utilizing the full pedigree is needed in order to target the origins of TRD.

In this study, after excluding previously known regions in Holsteins, 10 potential regions displayed |TRD|> 0.25 and ≥ 5,000 under-represented offspring and are summarized in Table 3. Three additional haplotypes and 2 SNPs were identified using the genotypic model as exhibiting recessive patterns (highly lethal in homozygous state), with ≥ 5,000 informative offspring and ≥ 45 non-observed homozygous offspring (Table 3). The 3 haplotype alleles with recessive patterns on BTA1 were physically close to each other (covering 10,696 Kbp) and showed similar TRD magnitudes, frequency and number of heterozygous sires, dams and non-observed homozygous offspring (Table 3), which potentially points to the same causal mutation (SNP, deletion, etc.). The LD between these 3 haplotypes ranged from 0.65 to 0.83. In addition, from 15,726 individuals carried a copy of the potential lethal allele in at least one of the 3 regions, 10,726 and 2,160 individuals carried a copy of the potential lethal allele in 3 and 2 regions simultaneously, respectively. This result gives extra evidence supporting the TRD found in this region.

**Table 3:** Potential new candidate regions with lethal alleles identified with transmission ratio distortion (TRD) in Holstein cattle

Chromo- some	Region (Kbp)	nSNP^a^	nº hetero^b^ sires	nº hetero dams	Frequency %	AB × AA^c^		AB × BB		AB × AB			TRD effects
						AA^d^	AB	AB	BB	AA	AB	BB	
1	27,285–27,325	2	79	1585	0.38	0	0	1204	8175	1	20	132	α = -0.37, α_s_ = -0.44, α_d_ = -0.30
1	102,167–103,035	20	188	4034	1.71	1	0	8213	8100	3	88	48	α_g_ = -0.57, δ_g_ = 0.29
1	109,285–109,707	10	196	4064	1.72	0	0	8557	8421	5	100	56	α_g_ = -0.56, δ_g_ = 0.29
1	112,498–112,863	10	190	3951	1.66	0	0	8467	8200	6	102	51	α_g_ = -0.54, δ_g_ = 0.29
3	20,502–20,970	7	60	743	0.50	0	0	2617	8036	11	37	26	α = -0.25, α_s_ = -0.28, α_d_ = -0.15
6	58,498–58,753	4	264	3500	1.63	0	0	6896	17674	49	290	298	α = -0.22, α_s_ = -0.27, α_d_ = -0.13
8	18,087–18,273	4	169	1825	0.87	0	1	4381	12313	37	105	98	α = -0.23, α_s_ = -0.30, α_d_ = -0.10
9	71,916	1^5^	211	1274	12.94	5	24	3397	3296	58	539	264	α_g_ = -0.47, δ_g_ = 0.25
9	77,576–77,734	2	265	3951	1.36	0	0	5441	18958	25	315	346	α = -0.27, α_s_ = -0.34, α_d_ = -0.17
10	42,238	1^5^	159	968	10.43	0	6	2477	2489	23	360	174	α_g_ = -0.56, δ_g_ = 0.28
12	147–742	10	29	154	0.43	0	0	2478	8157	2	9	11	α = -0.27, α_s_ = -0.28, α_d_ = -0.04
19	60,921–60,949	2	144	1885	0.76	0	0	3319	9703	12	63	68	α = -0.24, α_s_ = -0.32, α_d_ = -0.11
22	9,621	1	125	599	98.86	27101	3884	21	35	570	230	254	α = 0.36, α_s_ = 0.38, α_d_ = 0.23
24	48,165–48,420	7	64	1106	0.37	0	0	1666	10289	8	25	66	α = -0.36, α_s_ = -0.43, α_d_ = -0.16
29	34,677–34,697	2	127	1582	0.68	0	0	3461	10560	15	46	62	α = -0.25, α_s_ = -0.30, α_d_ = -0.15

α Overall TRD, α_s_ Sire-TRD, α_d_ dam-TRD, α_g_ Additive-TRD, δ_g_ Dominance-TRD

^a^Number of SNPs on the haplotype window

^b^Number of heterozygous sires

^c^Parents’ genotypes

^d^Offpsring genotype

^e^Raw data

### Functional analyses of candidate lethal allele regions

The positional candidate genes annotated in (i) the 100 Kbp interval downstream and upstream from the potential lethal SNPs and (ii) the genes mapped within the interval of the candidate lethal TRD haplotypes are shown in Supplementary Material 4. In total, 1,400 positional candidate genes were successfully uploaded in ingenuity pathway analysis (IPA) software. The canonical pathways and diseases and functions identified by IPA are shown in Supplementary Materials 5 and 6, respectively. Two canonical pathways were significantly enriched among the genes annotated in the potentially lethal TRD regions: nucleotide excision repair (NER) pathway (*p*-value = 0.025; genes = *HIST2H4A*, *POLE2*) and purine nucleotides de novo biosynthesis (*p*-value = 0.026; genes = *GMPS*). Genes implicated in NER pathway are involved in the process of DNA repair, NER eliminates structural DNA lesions, such as bulky, helix-distorting adducts [53]. DNA repair, specifically NER pathway, has been shown to be crucial for fertility, as this mechanism is essential to maintain the fidelity of DNA replication during mitotic, meiotic processes in both male and female germ cells [54, 55]. On the other hand, purine de novo biosynthesis has shown to be critical during early embryo preimplantation development in mouse embryos [56].

**Fig. 3:**
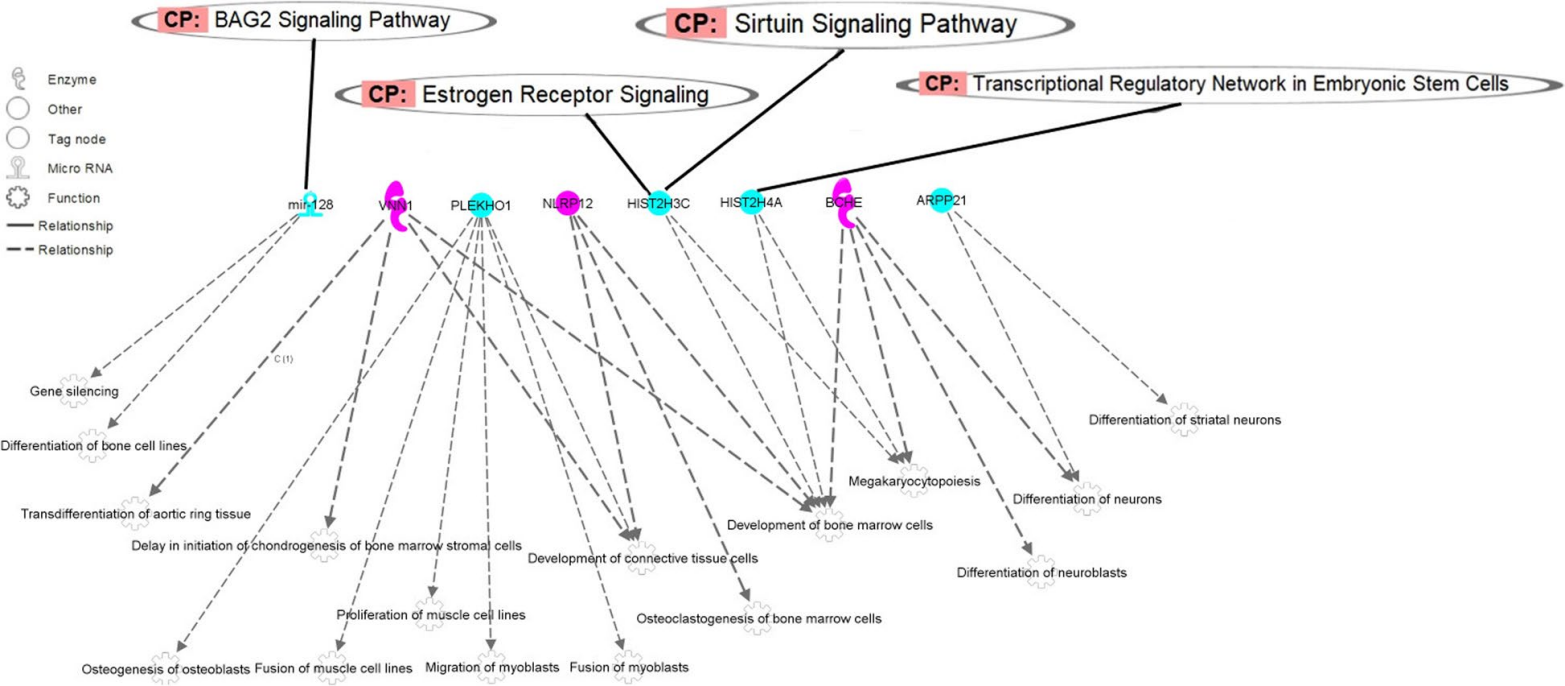
Network interaction between positional candidate genes on TRD regions and cellular development related canonical pathways and biological functions. The first layer corresponds to the canonical pathways, the second layer to the positional candidate genes and the third layer, the biological pathways. The colors of the positional candidate genes correspond to the observed TRD pattern, i.e. recessive TRD (in blue) and sire TRD (in pink)

A total of 178 enriched diseases were identified (*p*-value < 0.05; Supplementary Material 6). The main enriched diseases and functions, together with the canonical pathways, were represented in a network interaction between the positional candidate genes and the related terms. Two main networks were created (Figs. 2 and 3), highlighting a relevant number of genes associated with important biological processes associated with embryo development, cell survival and reproduction.

The first network, represented in Fig. 3, is composed by the genes *bta-mir-128*, *ARPP21*, *PLEKHO1*, *HIST2H3C*, *HIST2H4A*, *NLRP12*, *BCHE*, and *VNN1*. The last three genes were associated with TRD markers displaying a recessive TRD, while the other genes were associated with markers displaying a sire TRD. This network shows an interaction between genes and biological functions associated with the development and maintaining bone marrow cells, osteoclast and bone cell lines, and differentiation of neuronal cells. Among the genes located in regions with sire TRD, it is worthy to highlight the presence of *HIST2H3C* and *HIST2H4A* genes. The *HIST2H3C* and *HIST2H4A* genes codify for Histone Cluster 2 H3 Family Member C and Histone Cluster 2 H4 Family Member A, respectively. The exchange of histone-to-protamine in sperm chromatin remodeling is a key step for fertilization, because this process determines the degree of chromatin condensation [57]. Among the genes located in regions identified showing recessive TRD, the *VNN1* gene codifies a glycophosphoinositol-anchored glycoprotein highly expressed in the Sertoli cells showing dysmorphic expression between male and female gonadal cells, indicating a role in the mammalian sexual development [58]. Additionally, *VNN1* plays a crucial role in the regulation of chondrogenesis [59].

The *NLRP12* gene encodes for the NLRP protein 12 (nod-like receptors with a pyrin domain). Although this protein family has a major role in innate immunity, there are several studies in the last decade that highlight their importance in oocytes and early embryos [60, 61]. Lastly, the *BCHE* gene encodes for butyrylcholinesterase enzyme. Studies in humans suggested that impaired activity of butyrylcholinesterase in the uterus may increase uterine motility and contraction and decrease fertility [62]. Moreover, butyrylcholinesterase activity has shown to be reduced in humans with abnormal seminal parameters, such as sperm count and mobility [63].

**Fig. 4:**
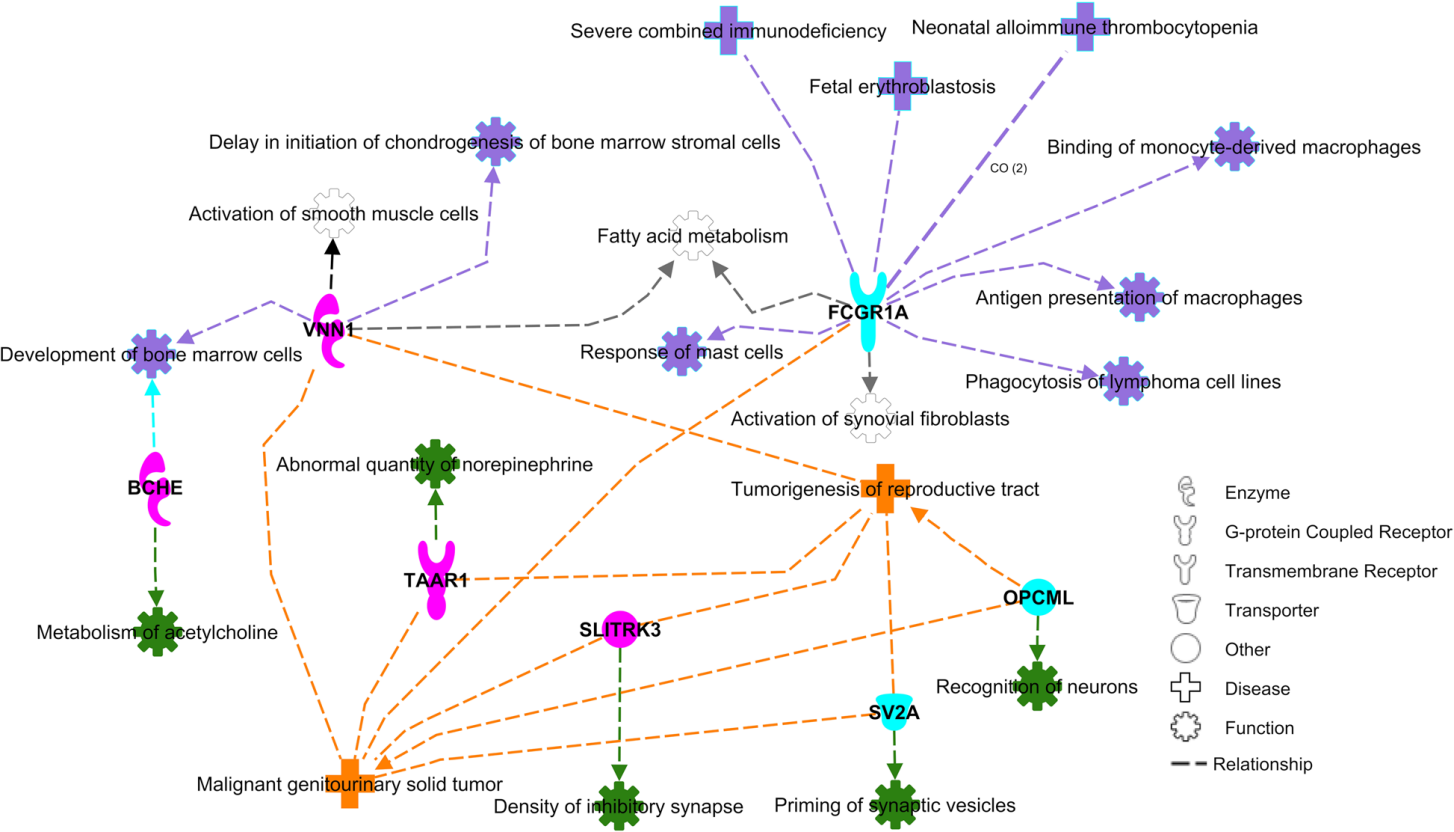
Network interaction between positional candidate genes on TRD regions and cell to cell interactions. The colors of the positional candidate genes correspond to the observed TRD pattern, i.e. recessive TRD (in blue) and sire TRD (in pink). The diseases and biological functions associated with the positional candidate genes were colored based on the functional similarity. The processes associated with immune response were colored in purple, the processes associated with the development of the nervous system was colored in green, and the processes associated with fertility were colored in orange

The second interaction network (Fig. 4) is composed of the genes *BCHE*, *TAAR1*, *VNN1*, *SLITRK3*, *SV2A*, *OPCML*, and *FCGR1A*. The first four genes are associated with markers displaying a recessive TRD pattern, while the last three genes are associated with markers displaying a sire TRD pattern. The biological functions showed in this interaction network are associated with immune response (purple), development of the nervous system (green), and fertility (orange). Among the genes in this network, the *SLITRK3* gene has been shown to be upregulated by the transcription factor gene cluster *RHOX* (X-linked reproductive homeobox). The *RHOX* gene cluster is expressed mainly in reproductive tissues and is known to have key roles in male fertility in mice and human, which suggest that *SLITRK3* may be one of the genes involved in the reproductive functions promoted by *RHOX * [64]. The *FCGR1A* gene encodes Fc fragment of IgG receptor Ia, which plays an important role in the immune response. A recent study evaluating the transcriptome of corpus luteum in sheep have highlighted the importance of immune system during early pregnancy, being *FCGR1A* one of the genes upregulated in high prolificacy sheep [65].

## Conclusions

Our study aimed to elucidate the prevalence of biased allele transmission in Holstein cattle. The results revealed the importance of implementing different TRD parameterizations to capture all types of distortions and to determine the corresponding inheritance patterns. The genotypic model highlighted alleles with classical recessive patterns, whereas the allelic model highlighted chromosomal regions with complete or quasi-complete absence for homozygous individuals and an under-representation (reduced viability) of the carrier heterozygous offspring as well. The full characterization of the genome allowed the identification of 604 chromosomal regions. Among them, the number of most relevant novel regions with strong allelic and recessive TRD patterns was 10 and 5, respectively. Additionally, the detection of TRD on previously published regions harboring recessive lethal alleles validated the TRD approach. Finally, novel candidate genomic regions containing lethal alleles and genes with functional and biological consequences on fertility and pre- and post-natal viability were also identified, which provides opportunities for improving breeding success in cattle.

## Methods

### Genotypes and trios

The dataset used in this study consisted of 441,802 Holstein genotypes from the Canadian Dairy Network (CDN) database (Lactanet, Guelph, ON). These animals were sampled from all available Holstein genotypes (> 1 million; October 2017), combining trios (sire-dam-offspring) with offspring genotyped within 90 days of birth, thus minimizing pre-selection of offspring [19, 66]. The total number of animals genotyped in trios were 340,363. The majority of trios (330,749) was structured in large paternal half-sib families with 10 to 5,725 offspring. The number of sires with at least 100 or 500 offspring was 572 and 150 with a total of 280,292 and 190,555 offspring, respectively. The animals of investigated trios, sires (*n* = 5,976), dams (*n* = 132,282) and offspring (*n* = 340,363) were genotyped with 22 different SNP genotyping arrays ranging from 2,900 to 777,962 SNPs (Table 4) and mapped to the UMD3.1 *Bos taurus* genome assembly. Given the different genotyping arrays, the number of available trios is different across SNPs. Only SNP markers with at least 10 trios were selected for TRD analyses using the original genotypes (raw-data, see Statistical Analyses section), resulting in a total of 132,991 autosomal SNPs distributed across the whole genome with different sample sizes of trios (from 10 to 340,363).

**Table 4:** Number of animals genotyped for each density of the SNP arrays

SNP array	Number of SNPs	Number of individuals
BovineHD BeadChip^a^	777,962	19
GGP Bovine 150 K Array^c^	139,914	8,513
Genomic Profiler-HD^c^	77,068	19,920
Medium Density V.2^d^	60,914	2,111
Bio-Gensys BGBoviSN^b^	57,513	28
Medium Density^d^	56,955	560
BovineSNP50 BeadChip^a^	55,647	43,710
GGP Bovine 50K^c^	47,850	4,328
Genomic Profiler LD V.4^c^	30,112	29,665
Low Density V.5^d^	27,780	6,589
Genomic Profiler LD^c^	26,151	62,091
Genomic Profiler-Super LD^c^	19,809	41,469
Low Density V.4^d^	18,815	52,003
Low Density V.2^d^	17,619	61,572
Low Density^d^	11,404	19,711
EuroG10K^a^	9,072	106
GGP Bovine 9K^c^	8,984	1,846
Genomic Profiler^c^	8,762	23,454
GGP Bovine 7K^c^	7,083	9,249
BovineLD BeadChip V.1.1^a^	6,912	4,529
BovineLD BeadChip^a^	6,909	36,907
Bovine3K BeadChip^a^	2,900	13,422

^a^Illumina, Inc., San Diego, CA

^b^Affymetrix, Santa Clara, CA

^c^Gene Seek, Lincoln, NE

^d^Zoetis, Florham Park, NJ

### Imputation to BovineSNP50 array

A total of 43,710 animals genotyped with the BovineSNP50 BeadChip (55,647 SNPs; Illumina, Inc., San Diego, CA) were used as a reference for imputation. The total number of autosomal SNPs was 47,910, after excluding SNPs with low genotype call rate (< 90%; map file in Supplementary Material 7). In order to ensure a high accuracy for imputation, we excluded animals genotyped with the lowest density (i.e., Illumina Bovine3K BeadChip (2,900 SNP)). Thus, the number of genotyped trios reduced to 283,817. The number of parents in the imputed data were 5,224 and 117,316 for sires and dams, respectively. The total number of genotyped animals was 373,793, which were imputed and phased using FImpute [67] with the option for population and family (pedigree) imputation to provide a more accurate imputation. The 47,910 imputed SNP genotypes were then used in the TRD analyses (imputed-data, see Statistical Analyses section).

#### Analytical models of transmission ratio distortion

##### Allelic parameterization of TRD

As described by Casellas et al. [15, 16], the probability of allele transmission (P) from heterozygote parents (A/B) to offspring was parameterized either including one overall TRD effect (α) on a parent-unspecific model or differentiating between sire- (α_s_) and dam-specific (α_d_) TRD effects on a parent-specific model:

$$\mathrm{P}(\mathrm{A}) = 1 -\mathrm{ P}(\mathrm{B}) = 0.5 + \alpha\ \mathrm{and\ P}(\mathrm{B}) = 1 -\mathrm{ P}(\mathrm{A}) = 0.5 -{ \alpha },$$

$${\mathrm{P}}_{\mathrm{i}}(\mathrm{A}) = 1 - {\mathrm{P}}_{\mathrm{i}}(\mathrm{B}) = 0.5 + {{\alpha }}_{\mathrm{i}}\mathrm{and}\ {\mathrm{P}}_{\mathrm{i}}(\mathrm{B}) = 1 - {\mathrm{P}}_{\mathrm{i}}(\mathrm{A}) = 0.5 - {{\alpha }}_{\mathrm{i}}\mathrm{ with\ i}= [\mathrm{s U d}]$$

where; α, α_s_ and α_d_ are TRD parameters which assumed flat priors within a parametric space ranging from -0.5 to 0.5. Under a Bayesian implementation, the conditional posterior probabilities of the TRD parameters are defined as:

$$\mathrm{p}({\alpha }|\mathbf{y}) \propto \mathrm{ p}(\mathbf{y}|{\alpha })\mathrm{p}({\alpha })\mathrm{\ and\ p}({{\alpha }}_{\mathrm{s}},{{\alpha }}_{\mathrm{d}}|\mathbf{y}) \propto \mathrm{ p}(\mathbf{y}|{{\alpha }}_{\mathrm{s}},{{\alpha }}_{\mathrm{d}})\mathrm{p}({{\alpha }}_{\mathrm{s}})\mathrm{p}({{\alpha }}_{\mathrm{d}})$$

where **y** is the column vector of genotypes of the offspring generation.

##### Genotypic parameterization of TRD

As developed by Casellas et al. [17], genotypic parameterization can be modeled by assuming additive (α_g_) and dominance (or over- / under-dominance,δ_g_) parameters, regardless of the origin of each allele. Following Casellas et al. [23], the probability of the offspring (P_off_) from heterozygous-by-heterozygous mating are:

$$\begin{aligned} {\mathrm{P}}_{\mathrm{off}}\left(\mathrm{AA}\right)=\frac{\left(1+{{\alpha }}_{\mathrm{g}}-{\updelta }_{\mathrm{g}}\right)}{4}, {\mathrm{P}}_{\mathrm{off}}\left(\mathrm{AB}\right)=\frac{\left(1+{\updelta }_{\mathrm{g}}\right)}{2}\\\mathrm{\ and }{\mathrm{\ P}}_{\mathrm{off}}\left(\mathrm{BB}\right)=\frac{\left(1-{{\alpha }}_{\mathrm{g}}-{\updelta }_{\mathrm{g}}\right)}{4}\end{aligned}$$

where; α_g_ and δ_g_ are additive- and dominance-TRD parameters, respectively. For heterozygous-by-homozygous mating, correction for overall losses of individuals in terms of genotypic frequency are needed to guarantee P_off_(AA) + P_off_(AB) + P_off_(BB) = 1. Thus, genotypic frequencies in offspring from AA × AB mating as an example become:

$$\begin{aligned} {\mathrm{P}}_{\mathrm{off}}\left(\mathrm{AA}\right)=\frac{\left(1+{{\alpha }}_{\mathrm{g}}-{\updelta }_{\mathrm{g}}\right)}{2\times (1+{{\alpha }}_{\mathrm{g}}/2)}, {\mathrm{P}}_{\mathrm{off}}\left(\mathrm{AB}\right)=\frac{\left(1+{\updelta }_{\mathrm{g}}\right)}{2\times (1+{{\alpha }}_{\mathrm{g}}/2)}\\ \mathrm{\ and\ }{\mathrm{P}}_{\mathrm{off}}\left(\mathrm{BB}\right)=0\end{aligned}$$

Under a Bayesian implementation, the conditional posterior probabilities of the TRD parameters are defined as:

$$\mathrm{p}({{\alpha }}_{\mathrm{g}},{\updelta }_{\mathrm{g}}|\mathbf{y}) \propto \mathrm{ p}(\mathbf{y}|{{\alpha }}_{\mathrm{g}},{\updelta }_{\mathrm{g}})\mathrm{p}({{\alpha }}_{\mathrm{g}})\mathrm{p}({\updelta }_{\mathrm{g}}|{{\alpha }}_{\mathrm{g}})$$

where **y** is the column vector of genotypes of the offspring generation. Flat priors were assumed for both α_g_ and δ_g_ within an extended parametric space. The initial range of the parametric space for α_g_ was [-1, 1] with a p(α_g_) = ½ and became restricted to [-1 + δ_g_, 1- δ_g_] with a p(α_g_) = 2 / (2—2 × δ_g_) when δ_g_ > 0. For δ_g_, the parametric space range was [-1, |α_g_|] with a p(δ_g_) = 1/(1 + α_g_).

##### TRD implementation on haplotype windows

To minimize random TRD and genotyping errors, the biallelic-haplotype procedure described by Id-Lahoucine et al. [19] was implemented to perform haplotype analyses. Assuming no specific interaction between alleles on parental generation under the same previously described parameterization for SNP markers, TRD parameters for each haplotype (H_j_) are generalized to:

$$\mathrm{P}({\mathrm{H}}_{\mathrm{j}}) = 1 -\mathrm{ P}({\mathrm{H}}_{-\mathrm{j}}) = 0.5 + {{\alpha }}_{\mathrm{j}}$$

$${\mathrm{P}}_{\mathrm{s}}({\mathrm{H}}_{\mathrm{j}}) = 1 - {\mathrm{P}}_{\mathrm{s}}({\mathrm{H}}_{-\mathrm{j}}) = 0.5 + {{\alpha }}_{\mathrm{sj}}\ \mathrm{and}\ {\mathrm{P}}_{\mathrm{d}}({\mathrm{H}}_{\mathrm{j}}) = 1 - {\mathrm{P}}_{\mathrm{d}}({\mathrm{H}}_{-\mathrm{j}}) = 0.5 + {{\alpha }}_{\mathrm{dj}}$$

where; H_j_ is the particular *j* haplotype under analyses, H_-j_ are the remaining alleles excluding *j*, α_j_, α_sj_ and α_dj_ are the overall, sire- and dam-specific TRD for the specific haplotype *j*, respectively.

The same strategy was implemented for the genotypic model, where from a heterozygous-by-heterozygous mating the probabilities of the offspring become:

$$\begin{array}{c}{\mathrm{P}}_{\mathrm{off}}\left({\mathrm{H}}_{\mathrm{j}}{\mathrm{H}}_{\mathrm{j}}\right)=\frac{\left(1+{{\alpha }}_{\mathrm{gj}}-{\updelta }_{\mathrm{gj}}\right)}{4}, {\mathrm{P}}_{\mathrm{off}}\left({\mathrm{H}}_{\mathrm{j}}{\mathrm{H}}_{-\mathrm{j}}\right)=\frac{\left(1+{\updelta }_{\mathrm{gj}}\right)}{2}\mathrm{\ and}\\ {\mathrm{P}}_{\mathrm{off}}\left({\mathrm{H}}_{-\mathrm{j}}{\mathrm{H}}_{-\mathrm{j}}\right)=\frac{\left(1-{{\alpha }}_{\mathrm{gj}}-{\updelta }_{\mathrm{gj}}\right)}{4}\end{array}$$

where; α_gj_ and δ_gj_ are additive- and dominance-TRD parameters for the specific haplotype *j*, respectively.

### Statistical analyses

Transmission ratio distortion was evaluated SNP-by-SNP across 132,991 SNPs (raw-data) and 47,910 SNPs (imputed-data) and using a sliding windows haplotype approach of 2-, 4-, 7-, 10- and 20-SNP across 47,910 SNPs. The average distance in base pairs between adjacent SNPs in the imputed data was 52,248. The analyses were performed within a Bayesian framework using TRDscan v.1.0 software [19] with a unique Monte Carlo Markov chain of 110,000 iterations, where the first 10,000 iterations were discarded as burn-in. The statistical significance of TRD was evaluated using a Bayes factor [68]. Both allelic and genotypic parameterizations were compared using the deviance information criterion [24] to determine the goodness-of-fit and the inheritance pattern of each region. In order to optimize TRD analyses, TRD regions initially identified with at least one of the three models, were subsequently filtered following Id-Lahoucine et al. [19]. Firstly, a minimal number of informative parents (≥ 20 heterozygous sires and/or ≥ 100 heterozygous dams) were considered to minimize possible false TRD from genotyping errors. Secondly, regions with few heterozygous sires displaying full skewed transmission and completely explaining the observed TRD in the corresponding region, were discarded as potential genotyping errors. Third, the approximate empirical null distribution of TRD [19] at < 0.001% margin error was used in order to eliminate TRD generated by chance (i.e., gamete sampling). Subsequently, regions with a large credible interval for TRD effects (i.e., coefficient of variation > 20%) given the unstable convergence, were filtered out. Finally, in order to integrate all the results to obtain clear highlighted peaks of TRD across the whole genome, a non-parametric technique, known as kernel smoothing [69, 70] was applied. The smoothed estimate of BF for the i_th_ base pair (bp) within the range κ_1_ to κ_n_ was calculated using weighted Gaussian kernel (${\widehat{\mathrm{y}}}_{\mathrm{i}}=\sum_{\mathrm{j}=1}^{\mathrm{n}}\frac{1}{\sqrt{2{{\pi \sigma }}^{2}}}\mathrm{exp}\left(-\frac{{({\mathrm{k}}_{\mathrm{i}}-{\mathrm{k}}_{\mathrm{j}})}^{2}}{2{\upsigma }^{2}}\right)\times {\mathrm{BF}}_{\mathrm{j}}$), where σ is the bandwidth, (κ_i_—κ_j_) is the distance in base pairs, and n is the total number of TRD regions included. The choice of the bandwidth for smoothing is an important component of its implementation, and different values were tested: σ = 500,000, 2,000,000 and 5,000,000 bp.

### Functional analyses

Fifteen potential new candidate regions with lethal alleles were annotated using the R package: Genomic functional Annotation in Livestock for positional candidate Loci, also known as ‘GALLO’ [71]. The.gtf file corresponding to the bovine gene annotation from UMD 3.1 assembly and the.gff file with the QTL information from Animal QTL Database [72] were used for gene and QTL annotation, respectively. In order to map the genes around SNPs displaying significant TRD an interval of 100 Kilobase pairs (Kbp) upstream and downstream from the SNP coordinate was used. For those regions displaying significant TRD identified through the haplotype analyses, the positional candidate genes were annotated within the coordinates for the haplotype interval. These positional candidate genes were investigated regarding its functional profile through an enrichment analysis for Canonical pathways, diseases and functions using ingenuity pathway analysis (IPA) software (QIAGEN Inc., Fall Release 2019,http://www.ingenuity.com; [73, 74]. However, first, the R package biomaRt v. 2.40.5 [75] was used to obtain the bovine Ensembl ID, the Gene Symbol and the respective orthologous in humans and mouse for each positional candidate gene. Only the orthologous genes showing a similarity higher than 75% with the bovine annotated genes were retained as the input for the IPA software. A significance threshold of *p*-value < 0.05 was adopted to consider an enrichment for all the categories tested (Canonical pathways, diseases and functions).

## Supplementary Information

**Additional file 1:** **Supplementary Material 1.** Threshold for the approximate empirical null distribution of additive and dominance transmissionratio distortion (TRD) for different ranges of number of informative offspring.

**Additional file 2:** **Supplementary material 2.** Summary of the final 604 chromosomal regionsdeemed with TRD across the bovine genome.

**Additional file 3:** **Supplementarymaterial 3.** Recessive haplotypes identified within TRD regions.

**Additional file 4:** **SupplementaryMaterial 4.** Genes mapped within the interval of the candidate lethal TRDhaplotypes.

**Additional file 5:** **Supplementarymaterial 5.** Canonical pathways associated with the positional candidate genesmapped within TRD regions.

**Additional file 6:** **Supplementarymaterial 6.** Diseases and functions associated with the positional candidate genes mapped within TRDregions.

**Additional file 7:** **Supplementarymaterial 7.** Genetic markers and the respective genomic coordinates used to identifyTRD across the bovine genome.

## Data Availability

The data that support the findings of this study are available from Lactanet (Guelph, Canada) but restrictions apply to the availability of these data, which were used under research agreement for the current study, and so are not publicly available. Data are however available from the Angela Canovas upon reasonable request and with permission of Lactanet (Guelph, Canada).
