# Supplementary material for: Unravelling transmission ratio distortion across the bovine genome: identification of candidate regions for reproduction defects
Source: BMC Genomics. 2023 Jul 8;24:383. doi: 10.1186/s12864-023-09455-6 (PMC10329377; doi:10.1186/s12864-023-09455-6)
Supplement: Supplementary file 1 — Additional file 1: Supplementary Material 1. Threshold for the approximate empirical null distribution of additive and dominance transmissionratio distortion (TRD) for different ranges of number of informative offspring. [file 12864_2023_9455_MOESM1_ESM.pdf]

Supplementary Material 1. Threshold for the approximate empirical null distribution of additive and dominance transmission ratio distortion (TRD) for different ranges of number of informative offspring

	Number of informative offspring[1]	Threshold on the null distribution[2]				
		5.00%	1.00%	0.10%	0.01%	0.001%
|Additive-TRD|	10 – 15	0.5649	0.6800	0.8282	1.0000	1.0000
	16 – 25	0.5569	0.6700	0.8063	1.0000	1.0000
	26 – 50	0.5561	0.6647	0.7825	0.9510	1.0000
	51 – 100	0.5079	0.6206	0.7507	0.9510	1.0000
	101 – 250	0.4247	0.5594	0.7098	0.9510	1.0000
	251 – 1,000	0.2950	0.4431	0.6224	0.7742	1.0000
	1,001 – 5,000	0.1416	0.2380	0.4041	0.5567	0.7839
	5,001 – 20,000	0.0503	0.0731	0.1207	0.2385	0.3107
	20,001 – 40,000	0.0242	0.0353	0.0520	0.0730	0.0730
	40,001 – 60,000	0.0181	0.0308	0.0363	0.0464	0.0464
|Dominance-TRD|	10 – 15	0.5192	0.6440	0.7911	0.9573	1.0000
	16 – 25	0.4453	0.5692	0.6880	0.8896	1.0000
	26 – 50	0.3912	0.5065	0.6698	0.8513	1.0000
	51 – 100	0.3261	0.4288	0.6119	0.8096	1.0000
	101 – 250	0.2548	0.3450	0.5260	0.7141	1.0000
	251 – 1,000	0.1658	0.2441	0.3555	0.5785	0.7721
	1,001 – 5,000	0.0799	0.1274	0.2094	0.2878	0.5962
	5,001 – 20,000	0.0304	0.0437	0.0644	0.1332	0.1555
	20,001 – 40,000	0.0151	0.0219	0.0315	0.0452	0.0652
	40,001 – 60,000	0.0121	0.0167	0.0227	0.0278	0.0278

[1]Informative offspring: offspring from one or both heterozygous parents for the overall TRD and from the specific heterozygous parent for the parent-specific TRD. [2]Symmetric distribution with two tails, the threshold corresponds to the sum of both sides.
